# The Regents, the Superintendent of Education and the Merritt Bill

**Published:** 1903-03

**Authors:** 


### The Regents, the Superintendent of Education and the Merritt Bill.

IT IS important that those pupils not residing in high school districts should have equal opportunities for education with those who live within the jurisdiction of secondary instruction. The Merritt bill proposes that the state shall pay for the tuition in high schools of those who desire to avail themselves of that privilege and are not now entitled without payment of tuition fees. It is highly important that any moneys appropriated for such purpose shall be disbursed without any taint of partisanship. It follows as a logical sequence that any such appropriation should be distributed through the regents in order to reach the end desired with economy, and with impartial benefit to those who for whom intended. There can be no two opinions concerning these two points.

It is unfortunate that we have a dual responsibility at Albany regarding our educational system. It leads to misfits, to friction, to acrimony, and it should be changed without delay. The legislature can do it if it will and there is no doubt the governor would approve the unification of the state educational system. One responsible head for all our departments has been the advice, even the policy, of Governor Odell, and we have seen abundant proof that he is a consistent man.

The address of the regents to the governor, the legislature, and the people outlines a plan that is so simple, so complete, so just, and, withal, so needful, that we can discover no objection to its prompt adoption, and we look to the legislature to grant the petition on its part, since it must take the initiative.

Every physician is interested in the perfection of our educational methods and the administration of the whole system upon a non-partisan basis. We hope our readers who reside in this state will manifest a practical interest in the matter, and take it up with those members of the legislature whom they happen to know. A prompt free discussion of the subject is what is needed; with this once had there ought to be no difficulty in settling the vexed question, on a foundation that would remove our public schools from the arena of partisan politics, and keep them in safe hands for all time.

---

*The Trained Nurse and Hospital Review* for January gives a very interesting account of the graduation of the first class of nurses from the Mercedes Hospital School for Nurses at Havana, Cuba, November 8, 1902. Seven nurses were given their diplomas. President Palma and other government officials were present, the nurses receiving their diplomas and badges from the hands of the president.

---

It is reported upon authority that Ithaca is invaded with a typhoid epidemic; that on February 7 some 400 cases had appeared. The last census credited the city with a population of 13,136, hence there is a proportion of nearly 33 cases of fever to 1,000 of population. It would seem that such a condition ought to be impossible at this period, when the methods of the propagation of this disease are so well understood, and when, too, the ways of prevention are so clear.

The water supply of this scourged university town should be purged of its infection at once, no matter at what cost, and everything should be put in the highest sanitary condition. Students are reported to be leaving the university by hundreds, which is but natural. It is stated that the university authorities demand that the Waterworks Company shall construct a new system. The university owns $100,000 worth of the bonds of the company and is anxious to undertake the financiering of the improvements, which will cost about $150,000. Delay in the matter will be criminal.

---

To reopen the case of the assassin of President McKinley, with reference to a discussion of his mental condition, is neither wise nor profitable. The case was closed by the courts a year and a half ago, and any further discussion of it can but serve again to invite attention to an abhorrent chapter in American history that had better be closed forever. Let the memory of the great President remain with us as a sweet example, but let his assassin remain in that oblivion to which his diabolical act very properly consigned him!

---

The Public Health and Marine Hospital Service of the United States is taking appropriate action in regard to the plague invasion of the Pacific coast. The conference of the several state boards of health at Washington in January, under the auspices of Surgeon-General Wyman, produced a good effect. The authorities of California have learned the importance of frankness in dealing with the question, and the energetic action of the Mexican National Board of Health has resulted in an appreciable abatement of the epidemic of Mazatlan. The United States Consul at that port has advised the Washington government that there had been a total number of cases, to February 1, of 260, with 200 deaths.

